# Bone Health in Patients with Dyslipidemias: An Underestimated Aspect

**DOI:** 10.3390/ijms23031639

**Published:** 2022-01-31

**Authors:** Panagiotis Anagnostis, Matilda Florentin, Sarantis Livadas, Irene Lambrinoudaki, Dimitrios G. Goulis

**Affiliations:** 1 Unit of Reproductive Endocrinology, 1st Department of Obstetrics and Gynecology, Medical School, Aristotle University of Thessaloniki, 56429 Thessaloniki, Greece; dimitrios.goulis@gmail.com; 2 Department of Internal Medicine, University Hospital of Ioannina, School of Health Sciences, University of Ioannina, 45110 Ioannina, Greece; matildaflorentin@yahoo.com; 3 Endocrine Unit, Metropolitan Hospital, 18547 Athens, Greece; sarntis@gmail.com; 4 2nd Department of Obstetrics and Gynecology, Medical School, National and Kapodistrian University of Athens, 11528 Athens, Greece; ilambrinoudaki@hotmail.com

**Keywords:** dyslipidemia, hypercholesterolemia, bone mineral density, osteoporosis, fractures, statins

## Abstract

Beyond being aging-related diseases, atherosclerosis and osteoporosis share common pathogenetic pathways implicated in bone and vascular mineralization. However, the contributory role of dyslipidemia in this interplay is less documented. The purpose of this narrative review is to provide epidemiological evidence regarding the prevalence of bone disease (osteoporosis, fracture risk) in patients with dyslipidemias and to discuss potential common pathophysiological mechanisms linking osteoporosis and atherosclerosis. The effect of hypolipidemic therapy on bone metabolism is also discussed. Despite the high data heterogeneity and the variable quality of studies, dyslipidemia, mainly elevated total and low-density lipoprotein cholesterol concentrations, is associated with low bone mass and increased fracture risk. This effect may be mediated directly by the increased oxidative stress and systemic inflammation associated with dyslipidemia, leading to increased osteoclastic activity and reduced bone formation. Moreover, factors such as estrogen, vitamin D and K deficiency, and increased concentrations of parathyroid hormone, homocysteine and lipid oxidation products, can also contribute. Regarding the effect of hypolipidemic medications on bone metabolism, statins may slightly increase BMD and reduce fracture risk, although the evidence is not robust, as it is for omega-3 fatty acids. No evidence exists for the effects of ezetimibe, fibrates, and niacin. In any case, more prospective studies are needed further to elucidate the association between lipids and bone strength.

## 1. Introduction

Dyslipidemias, which encompass a wide range of disorders in lipoprotein metabolism, constitute one of the leading causes of atherosclerotic cardiovascular disease (ASCVD) worldwide, mainly coronary heart disease (CHD) [1]. Osteoporosis is another common entity characterized by deterioration of bone microarchitecture, compromising bone strength and leading to increased risk of fractures [2]. Besides being age-dependent degenerative processes, atherosclerosis and osteoporosis share common underlying pathogenetic mechanisms involving bone and vascular mineralization [3]. Briefly, these include estrogen, vitamin D and K deficiency, increased concentrations of parathyroid hormone (PTH), homocysteine and lipid oxidation products, increased oxidative stress, and several other metabolic pathways that promote both the atherosclerotic process and bone loss. However, the role of dyslipidemias in skeletal health is less documented, with many studies yielding inconsistent results.

The purpose of this narrative review is to provide epidemiological and pathophysiological evidence regarding the association between dyslipidemia and bone mass and fracture risk. The effect of available hypolipidemic agents on bone health is also discussed.

## 2. The Association between Dyslipidemia and Bone Mineral Density or Fracture Risk

### 2.1. Bone Mineral Density

In general, epidemiological data regarding the association between lipid profile and bone mineral density (BMD) are inconsistent. Although some studies have shown no association between dyslipidemia and BMD, others have reported either a negative or a positive effect for each lipid parameter [4,5,6,7,8,9,10,11,12,13,14,15,16,17,18,19,20,21,22,23,24]. A summary of the highly cited studies is presented in Table 1, illustrating their heterogeneity.

In brief, 712 women and 450 men (aged 32–61 years at baseline) in the seminal Framingham cohort were studied with serum lipid measurements every 2 years until 1988–1989, when bone densitometry was performed. No association was found between averaged total cholesterol (TC) and BMD at any of the assessed skeletal sites [total hip (TH), lumbar spine (LS), and distal radius] [9]. Survival bias is a possible reason for the absence of association since individuals with hyperlipidemia are at a higher risk of death compared to those with normal lipid profiles. This was also the case with another study, including 958 postmenopausal Korean women, which found no association between LS or femoral neck (FN) BMD with TC, low-density lipoprotein cholesterol (LDL-C), triglyceride (TG), or high-density lipoprotein cholesterol (HDL-C) concentrations, either at the beginning or at the end of follow-up (7.1 years). The study, however, could not account for the use of anti-osteoporotic medication, which may have influenced the results [18]. The absence of association between these lipid parameters with BMD was also supported by a Greek cross-sectional study (*n* = 591 postmenopausal women) [19]. Interestingly, the authors found a negative association with LS BMD only for lipoprotein (a) (Lp(a)) in women ≥53 years and for HDL-C for those <53 years [19]. As this study was based on a convenience sample of women attending a menopause clinic in a tertiary care hospital, selections biases cannot be excluded. A negative association between Lp(a) concentrations and LS or FN BMD was also shown in a cross-sectional study, including 52 overweight early postmenopausal women [8]. However, a post-hoc analysis from the Women’s Health Initiative (WHI) study (*n* = 9698 postmenopausal women) showed no association between Lp(a) concentrations and hip BMD [25].

Another study investigated the association between lipid profile and BMD in two different cohorts, one from the general population (*n* = 265 males, 481 females) and one from patients attending an osteoporosis clinic (*n* = 236 pre- and postmenopausal women) [7]. In both groups, TH BMD was positively associated with LDL-C (*p* < 0.05) and TG (*p* < 0.05) and negatively with HDL-C concentrations (*p* < 0.05) in both genders, even after adjustment for body weight, height, and fat mass. Total body and LS BMD were also positively associated with LDL-C and TG and negatively with HDL-C concentrations in the general population and the clinic cohort, respectively [7]. The positive association of TH BMD with TC/LDL-C and the inverse association with HDL-C was replicated in a large US cohort (*n* = 13,592 participants of the National Health and Nutritional Examination Survey (NHANES) III). Nevertheless, after correction for potential confounders, such as age, sex, body mass index (BMI), and statin use, the association between lipid profile and BMD lost significance [11].

In contrast, another cross-sectional study (*n* = 1303 postmenopausal women who attended a menopause clinic) showed a higher prevalence of low LS BMD in those with high LDL-C concentrations (>160 mg/dL) compared with those with LDL-C <160 mg/dL (47.9% vs. 21.2%, respectively) [5]. A Japanese study in postmenopausal women referred to a tertiary care clinic for the evaluation of osteoporosis also showed an inverse association between LDL-C and forearm BMD, including 214 postmenopausal women. LS BMD was marginally not associated with LDL-C (*p* = 0.051). On the other hand, HDL-C concentrations were positively correlated with BMD values in these sites [4].

Aiming to overcome these discrepancies, some meta-analyses were conducted. One meta-analysis that included ten studies [26] compared the lipid profile between postmenopausal women with osteoporosis and normal BMD. HDL-C and TC concentrations were higher in the osteoporosis compared with the normal BMD group [HDL-C: mean difference (MD) 2.63, 95% confidence interval (CI) 0.43–4.84; TC: MD 14.82, 95% CI 2.84–26.80]. LDL-C concentrations also tended to be higher in the osteoporosis group (LDL-C: MD 9.67, 95% CI from −0.10 to 19.44). No difference in TG concentrations between groups was observed.

Another recent meta-analysis [27] included data from 12 case-control studies in patients with osteopenia or osteoporosis (*n* = 12,395). It investigated the difference in lipid profile in comparison with individuals with normal BMD. Regarding TC, there was no difference between patients with low bone mass (osteopenia or osteoporosis) and controls, irrespective of gender or hypolipidemic medication. This was also the case regarding LDL-C and TG concentrations. LDL-C concentrations were higher only in the osteoporosis subgroup, which was not prescribed any lipid-lowering medication, compared with controls [(mean difference (MD) 0.16 mmol/L, confidence interval (CI) 0.02–0.31]. Moreover, TG concentrations were slightly higher in patients with osteopenia compared with controls only in the subgroup prescribed hypolipidemic treatment (MD 0.07 mmol/L, 95% CI 0.03–0.12). Concerning HDL-C concentrations, these were higher in patients with osteoporosis compared with those with normal BMD (MD 0.05 mmol/L, 95% CI 0.03–0.07), whereas there was no difference between osteopenia and control groups in this regard.

Few studies exist in children and adolescents concerning the association between lipid profile and BMD, yielding inconsistent results. In a cross-sectional study from China (*n* = 14,303; 49.9% boys; mean age 11.4 ± 3.3 years), an inverse association was observed in both sexes between serum TG concentrations and calcaneus BMD [28]. Similar results were shown in a cross-sectional study from Spain (*n* = 188 overweight/obese children; mean age 10.4  ±  1.2 years), in which metabolically unhealthy children with metabolic syndrome (including high TG and low HDL-C concentrations) presented lower areal BMD compared with metabolically healthy ones [29]. In contrast, a cross-sectional study from the UK (*n* = 2305; 47.7% males; mean age 15.5 years) showed a positive association between serum TG and total body BMD, bone mineral content, and bone area. HDL-C was inversely associated with these parameters [30]. Moreover, in a small, cross-sectional study (*n* = 47 Caucasian male adolescents; mean age 16.9 years), a positive association was observed between serum Lp(a) concentrations and femoral neck BMD [31]. However, in a cross-sectional study (*n* = 306 girls; mean age 10.8 ± 1.1 years), there was no association between lipid profile (TG, HDL-C, LDL-C) and total or areal BMD [32].

### 2.2. Dyslipidemia and Fractures

In general, little data exist concerning the association between dyslipidemia and fractures. According to a recent meta-analysis, which included ten studies (six prospective, three cross-sectional, and one case-control; 3–20 years of follow-up; *n* = 60,484 individuals; mean age >25 years), a 50 mg/dL increase in TC concentrations was associated with 15% higher risk of fractures (combined effect size 1.15, 95% CI 1.02–1.30). Concerning HDL-C, although there was no association with fractures, subgroup analysis from prospective studies showed an 18% lower risk of fractures in those with HDL-C < 40 mg/dL compared with individuals with HDL-C > 40 mg/dL (combined effect size: 0.82, 95% CI 0.71–0.96). Finally, no association between either TG or LDL concentrations and fracture risk was documented [33]. Similarly, no association exists between Lp(a) concentrations and fracture risk [25].

### 2.3. Critical Review of Available Data

The abovementioned data point towards a positive association between dyslipidemia (especially high TC and LDL-C concentrations) and low bone mass or fractures, although firm conclusions cannot be drawn. Most of these studies were cross-sectional in design or used a convenience sample. Furthermore, many confounders, such as dietary habits of calcium intake, alcohol or caffeine consumption, smoking, socioeconomic status, ethnicity, gender, exercise and physical activity, or comorbidities such as diabetes, which intervene with the association between lipid profile and bone mass, should be acknowledged. Another shortcoming of these studies is excluding patients with ASCVD prescribed lipid-lowering medication or at high risk of fracture prescribed anti-osteoporotic medication.

## 3. Pathogenetic Mechanisms Linking Dyslipidemia and Atherosclerosis with Impaired Bone Metabolism

### 3.1. Direct Effect of Dyslipidemia on Bones

Several, though not all, lines of evidence have shown an inverse association of BMD with TC and LDL-C [34]. It has been suggested that increased cholesterol inhibits osteoblast differentiation, preventing bone formation [35]. Enhanced osteoclastogenesis may also be involved [35]. A differential effect of serum cholesterol on BMD at various skeletal sites has been suggested [34], explaining the inconsistency among studies. Furthermore, low HDL-C concentrations have been associated with the development of an inflammatory microenvironment and increased bone marrow adiposity, which restrains the differentiation and function of osteoblasts, leading to reduced bone mass [36].

Bones and vascular tissue share similar pathological features. As with atherosclerosis, increased lipids accumulate beneath the vascular intima and perivascular space in bones [37]. Furthermore, inflammatory bioactive lipids, which promote atherosclerosis, also induce bone loss [37], whereas oxidized LDL-C appears to play a major role in bone loss [38]. Lipid oxidation products, such as minimally oxidized LDL-C, promote arterial calcification, possibly by activating osteoblasts in the arterial pool, while their accumulation in the subendothelial space of skeletal bone arteries inhibits bone formation [39]. Dyslipidemias are also associated with impaired nitric oxide (NO) and enhanced endothelin production, leading to endothelial cell dysfunction and increased thrombotic risk [40]. Additionally, isoprostanes, present in atherosclerotic plaques, enhance vasoconstriction and endothelin-1 release in endothelium and modulate platelet aggregation [41]. Isoprostanes also inhibit osteoblastic differentiation of pre-osteoblasts and enhance osteoclastic differentiation and activity [41]. Overall, lipids appear to be involved in bone remodeling and atherosclerosis progression in opposite directions, explaining the simultaneous existence of osteoporosis and atherosclerosis in people with dyslipidemia [42].

Other mechanisms involve fat accumulation in the femoral head, which increases bone marrow microcirculation pressure and reduces bone vascularization, resulting in ischemia and hypoxia [43]. Moreover, increased blood viscosity also compromises bones’ blood supply [43]. All these phenomena may lead to bone necrosis and fractures [43]. A summary of the direct and indirect mechanisms linking dyslipidemia with bone loss is provided in Table 2.

### 3.2. Indirect Mechanisms

#### 3.2.1. Estrogens

Bone and coronary arteries are target organs for estrogens. Indeed, estrogen receptors have been detected on osteoblasts, osteoclasts, and coronary artery smooth muscle cells [44]. Estrogen loss during the transition to menopause leads both to bone loss [45] and atherogenic dyslipidemia [46,47]. Reduced estrogen concentrations have also been associated with increased PTH secretion [48] and, subsequently, accelerated bone loss and soft tissue calcium deposition, including vascular and myocardial calcification [49]. Moreover, an inverse association between estrogen and serum homocysteine concentrations, as well as oxidized LDL-C, has been reported [3], which may partly explain the increased risk for both osteoporosis and atherosclerosis in menopause.

#### 3.2.2. Vitamin D and PTH

Vitamin D and PTH are associated with the regulation of phosphorus metabolism, and dysregulated phosphorus metabolism is associated with bone mineral disorders and vascular calcification [50]. Vitamin D deficiency leads to decreased calcium absorption from the intestine and calcium release from bones to maintain normal serum calcium concentrations [51]. Moreover, low vitamin D status increases fracture risk via secondary hyperparathyroidism, leading to bone demineralization and the development of osteomalacia and osteoporosis [51]. On the other hand, vitamin D receptors (VDR) are present in endothelial and smooth muscle cells of the arterial wall [3,52]. Some, but not all, studies suggest that polymorphisms of VDR may be involved in the mutual risk between osteoporosis and atherosclerosis [3,52]. Moreover, increased PTH concentrations are associated with an increased risk of ASCVD [53].

#### 3.2.3. Systemic Inflammation

Inflammation is an important component in the pathogenesis of atherosclerosis and osteoporosis, and inflammatory rheumatic diseases have been associated with secondary atherosclerosis and increased bone loss [54]. Several inflammatory mediators are involved in both clinical entities. Furthermore, high concentrations of some inflammatory mediators [e.g., tumor necrosis factor α (TNF-α), interleukin (IL)-1, IL-6, IL-17, and C-reactive protein] have been associated with increased risk of myocardial infarction and non-traumatic fragility fractures [53].

#### 3.2.4. Carboxyglutamic Acid (Gla) Proteins

Gla proteins, including matrix Gla protein (MGP) and osteocalcin, encompass a part of a family of mineral-binding proteins. Gla residues bind and incorporate calcium into hydroxyapatite crystals [55]. MGP is a secretory protein with widespread tissue expression, including bones and vascular walls, inhibiting the osteoid formation and mineralization [56,57]. Osteocalcin, an abundant protein in bones, also inhibits calcification. Data from animal studies suggest that depletion or dysregulation of these proteins leads to abnormal mineralization of bones and arteries [56]. Regarding humans, MGP is integrally expressed in the normal aorta, while it is up-regulated in atherosclerotic plaques [58], probably to limit vascular osteogenesis. Osteocalcin resembles MGP expression in normal and atherosclerotic human vessels [59]. Indeed, increased serum osteocalcin concentrations have been observed in women with both atherosclerosis and osteoporosis [60,61].

#### 3.2.5. Vitamin K

Vitamin K is an essential co-factor for the formation of Gla proteins. Accordingly, reduced availability of vitamin K has been associated with functionally defective Gla proteins, which cannot properly form the matrix in which calcium and phosphorus bind together to make solid, well-mineralized bone; thus, low BMD ensues [62]. On the other hand, impaired vitamin K status has been associated with the presence of atherosclerotic calcification [3]. A possible underlying mechanism is that, by increasing MGP in the arterial wall, vitamin K protects against the calcification induced by osteocalcin [62].

#### 3.2.6. Osteopontin

Osteopontin (OPN) is an extracellular, non-collagenous bone matrix glycoprotein, which binds to integrins, especially the αvβ3 one. Integrins are transmembrane proteins that facilitate cell–cell and cell–extracellular matrix adhesion [63]. In bone, upon OPN binding, integrin αvβ3 activates signal transduction pathways that mediate osteoclast attachment to resorption sites [55,64], subsequently promoting bone resorption [64,65,66]. Apart from bones, OPN appears to be involved in vascular inflammation and atherosclerosis [67]. Several lines of evidence suggest that OPN promotes atherosclerotic plaque formation, leading to artery calcification [68,69,70]. In patients with CHD, OPN was found to be localized in calcified atherosclerotic lesions [59] and calcified cardiac valves [71]. Several possible underlying mechanisms regarding the association of OPN expression with increased atherosclerotic risk have been proposed, such as enhanced endothelial cell migration via αvβ3 ligand, increased macrophage activation, and cytokine release by OPN [72,73].

#### 3.2.7. Bone Morphogenetic Proteins

Bone morphogenetic proteins (BMPs) belong to the transforming growth factor β (TGF-β) superfamily. TGF-β exerts a fundamental role in regulating osteoblast differentiation and proliferation [74,75]. Among BMPs, BMP-2 and BMP-7 promote collagen synthesis and are involved in bone and cartilage formation [76]. Besides its role in bone homeostasis, BMP signaling has also been well described in endothelial cells [77]. Furthermore, megakaryocytes and platelets also contain BMP-2 and BMP-4 [78], the latter being a mediator of vascular inflammation in early atherosclerosis and restenosis [79].

Considering that BMPs induce bone formation and that BMP-2 is expressed by vascular endothelial and smooth muscle cells, it may be assumed that the vascular expression of BMPs could favor calcification. Indeed, increased BMP-2 and Cbfa-1 have been demonstrated in human atherosclerotic lesions, whereas this does not occur in normal arteries [80]. Furthermore, BMP-2 expression in the arterial wall is regulated by proinflammatory stimuli, such as TNF-α and oxidized lipids, hyperglycemia, and maybe a feature of atherosclerotic calcification [81].

#### 3.2.8. Homocysteine

Homocysteine is involved in bone metabolism via several mechanisms. Overall, it fosters osteoclastogenesis and increases osteoclast activity, subsequently enhancing bone resorption, while it decreases osteoblast activity and delays the synthesis of complex cross-links in collagen [82]. Of note, hyperhomocysteinemia appears to be a marker for increased risk of osteoporosis and osteoporotic fractures, as it reduces blood supply in the bone and may influence the biomechanical properties of bones [83]. However, data regarding the link between homocysteine and osteoporosis are inconsistent [84,85,86]. On the other hand, hyperhomocysteinemia has also been associated with premature atherosclerosis and thromboembolism. Several underlying mechanisms have been proposed, such as endothelial dysfunction, diminished NO bioavailability, lipid peroxidation, smooth muscle cell proliferation, and increased platelet aggregation [87,88]. Of note, dyslipidemia (mainly hypertriglyceridemia and low HDL-C concentrations) is positively associated with serum homocysteine concentrations [89].

#### 3.2.9. Nitric Oxide

NO has atheroprotective properties. Briefly, endothelial NO promotes vascular smooth muscle relaxation and inhibits platelet aggregation and adhesion, as well as LDL-C oxidation [90,91,92]. NO is also produced in bone cells by all three NO synthase (NOS) isoforms, i.e., endothelial, neural, and inducible NOS [93]. Concerning bone metabolism, low physiological NO concentrations stimulate bone formation and fracture healing, whereas pathologically, high NO concentrations inhibit these processes [94]. Consistently, animal studies suggest that the biological response to NO is dose-dependent in bone and vascular tissue, with NOS accentuating bone loss and NOS deficiency accelerating atherosclerosis and osteoporosis [42].

#### 3.2.10. RANK/RANKL/OPG System

The receptor activator of nuclear factor kappa-Β (RANK)/RANK ligand (RANKL)/osteoprotegerin (OPG) system plays a major role in maintaining the coupling between bone resorption by osteoclasts and bone formation by osteoblasts [95]. RANKL is released by osteoblast lineage cells and binds to RANK, leading to the differentiation of osteoclast precursor cells into mature osteoclasts [96]. OPG is a soluble glycoprotein, largely expressed in osteoblast lineage cells and various other cell types, including vascular endothelial cells [97]. OPG, as a decoy receptor for RANKL, inhibits RANK–RANKL interactions and subsequently osteoclastogenesis and bone resorption [98]. On the other hand, the upregulation of the RANK/RANKL/OPG axis has been associated with arterial and valve calcification [99].

#### 3.2.11. Wnt Pathway

The Wingless-related integration site (Wnt) pathway, which comprises a large family of 19 secreted signaling glycoproteins and 10 frizzled receptors [100], is involved in regulating osteoblastogenesis and bone formation. These glycoproteins bind to receptor complexes, such as LDL receptor-related proteins (LRP)-5 and LRP-6 [101], and stimulate gene expression associated with bone development [102]. Accumulating evidence suggests that Wnt signaling improves intracellular cholesterol trafficking through mechanisms other than the classical LDL receptor pathway. Of note, Wnts have been demonstrated to protect against atherosclerosis and coronary artery occlusion [103]. Furthermore, Wnt ligands appear to regulate the pathologic calcification in the vasculature and the osteoblastic trans-differentiation of smooth vascular cells in vitro [104]. Wnt signaling antagonism by oxidative stress may be one of the underlying mechanisms associated both with osteoporosis and atherosclerosis, as well as impaired glucose and lipid metabolism [105].

Sclerostin, an inhibitor of Wnt-mediated osteoblast activation and bone formation, is also involved in bone resorption and subsequent BMD reduction [106] and is associated with early atherosclerosis [107].

#### 3.2.12. Cbfa1 and Runx-2 Transcription Factors

The transcription factors core-binding factor alpha 1 (Cbfa1) and runt-related transcriptional factor-2 (Runx-2) promote osteoblastic differentiation at the early stage but inhibit it at the late stage [3,108]. These factors enhance the expression of bone matrix protein genes and the mineralization in immature mesenchymal and osteoblastic cells in vitro [3,108]. Moreover, Runx-2 expression has been identified in atherosclerotic human vascular tissue specimens. Specifically, oxidative stress likely induces vascular smooth cell calcification by modulating Runx-2 [109].

## 4. The Effect of Hypolipidemic Medications on Bone Metabolism

### 4.1. Statins

The 3-hydroxy-3-methylglutarylo-CoA (HMG-CoA) reductase inhibitors (statins) are the most widely prescribed lipid-lowering agents, constituting the mainstay of treatment both in adults and children with hypercholesterolemia [110,111]. Preclinical and clinical data suggest a potential beneficial effect on bone metabolism. In particular, statins may inhibit osteoclastic activity since HMG-CoA blockade reduces the production of downstream products in the mevalonate pathway, such as farnesyl pyrophosphate (FPP) and geranylgeranyl pyrophosphate (GGPP) [112]. The same pathway is also shared by nitrogen-containing bisphosphonates, thus preventing the prenylation of guanosine triphosphate (GTP)-ases, such as Ras, Rho, and Rac, which are essential for the survival and function of osteoclasts [113]. Another mechanism could be the inhibition of RANKL, which is essential for osteoclast differentiation by averting the production of reactive oxygen species [114]. Statins may also increase 25-hydroxy-vitamin D concentrations [52].

Statins also exert osteoanabolic properties, inhibiting osteoblast apoptosis and fostering osteoblast activity. This mechanism is mediated through increased expression of the *BMP-2* gene, which promotes osteoblast differentiation [115]. The latter is also induced by the depletion of FPP and GGPP, as mentioned above [116]. Statins may also promote embryonic stem cell differentiation towards the osteogenic lineage, through activation of increased mRNA expression of runt-related gene 2 (*Runx2*), osterix (*OSX*), and osteocalcin (*OCN*), as osteogenic transcription factors [117].

However, data in humans regarding the effect of statins on bone mass and, more importantly, fracture risk are not robust and consistent. A meta-analysis of randomized controlled trials (RCTs), published in 2016, including seven studies involving a total of 27,900 subjects, showed an increase in BMD by 0.03 g/cm^2^ (95% CI 0.006–0.053; I^2^ 99.2%; *p* < 0.001) with statins. Concerning the skeletal site, four studies assessed BMD in LS, one in the distal radius and two in any of multiple skeletal sites. Regarding fracture risk, no association with statin use was observed [pooled hazard ratio (HR) 1.00, 95% CI 0.87–1.15; I^2^ 0; *p* = 0.396]. These findings remained consistent and significant in sensitivity analysis [118].

These results were replicated by another meta-analysis published in 2017, including 33 studies (23 observational and ten RCTs) with 314,473 patients on statin therapy and 1,349,192 controls [119]. In particular, statins increased LS BMD (standardized MD (SMD) 0.20, 95% CI 0.07–0.32; *p* = 0.002; I^2^ 43%), as well as TH BMD (SMD 0.18, 95% CI 0.00–0.36; *p* < 0.05; I^2^ 62%). In subgroup analyses, these associations remained significant only for data derived from cohort studies but not for RCTs. Notably, there was no gender difference regarding TH, but LS BMD increased only in males. Concerning FN BMD, no association with statin use was found. Regarding fracture risk, statins decreased the risk of overall (odds ratio (OR) 0.81, 95% CI 0.73–0.89; *p* < 0.0001; I^2^ 87.5%) and hip fractures (OR 0.75, 95% CI 0.60–0.92; *p* = 0.007; I^2^ 77.2%), however, with no effect on vertebral and upper extremity fractures (data from 16 cohort and case-control studies). The authors also assessed the effect of statins on markers of bone turnover, showing a positive effect on osteocalcin concentrations (SMD 0.21, 95% CI 0.00–0.42; *p* = 0.04; I^2^ = 0%), but no effect on bone-specific alkaline phosphatase (bALP) and serum C-terminal peptide of type I collagen (CTX) concentrations [119].

Interestingly, a recent Mendelian randomization (MR) study showed that the effect of statins on BMD was dependent on the degree of their LDL-C-lowering action. MR explained this by utilizing 400 single nucleotide polymorphisms, which provided evidence for a causal effect of LDL-C on BMD [120].

Another recent meta-analysis assessed the effect of statin use exclusively on fracture risk in older adults [121]. The authors included 21 observational studies and two RCTs (*n*= 1,783,123 participants). Data from the observational studies showed an overall decreased fracture risk with statin use [pooled relative risk (RR) 0.80, 95% CI 0.72–0.88; I^2^ 93.1%]. In subgroup analysis, this association was more evident in men (RR 0.75, 95% CI 0.59–0.95) than in women (RR 0.87, 95% CI 0.76–0.99) and only for hip (RR 0.73, 95% CI 0.64–0.82) and low extremity fractures (RR 0.69, 95% CI 0.54–0.88). In terms of statin type, only atorvastatin was associated with a reduction in fracture risk (RR 0.77, 95% CI 0.71–0.84) compared to other statins. Interestingly, this beneficial effect was shown only for a short duration of statin use (<1 year) (RR 0.66, 95% CI 0.47–0.93), but not for a higher duration (1–3 or >3 years). Of note, it must be emphasized that the evidence for an anti-fracture efficacy for statins was based only on data derived from observational studies. In the two RCTs, there was no evidence for reducing fracture risk with statin use (RR 1.00, 95% CI 0.87–1.15; I^2^ 0%) [121].

### 4.2. Ezetimibe

Ezetimibe acts by blocking the cholesterol transport protein Nieman-Pick C1-like 1 (NPC1L1) protein, inhibiting the intestinal absorption of cholesterol. This increases the expression of the LDL-C receptor in hepatocytes, resulting in reductions in serum LDL-C concentrations by 20% [122]. However, due to the concomitant upregulation of cholesterol biosynthesis, mevalonate concentrations may increase. Scarce data exist concerning its effect on bone metabolism. In particular, the inhibition of NPC1L1 protein has raised the hypothesis of reduced vitamin D absorption since NPC1L1 is an important sterol transporter [123]. Although experimental data have shown a decrease in 25(OH)D concentrations [124], this has not been shown in human studies [125]. Moreover, in an open-label study (*n* = 54 patients with hypercholesterolemia), no effect in LS and TH BMD, as well as in bone turnover markers (bALP and CTX), was observed with ezetimibe [126].

### 4.3. PCSK-9 Inhibitors

No data concerning the effect of proprotein convertase subtilisin/kexin type 9 (PCSK-9) inhibitors on bone metabolism are currently available.

### 4.4. Fibrates

Fibrates are proliferator-activated receptor (PPAR)-α agonists, mostly used in patients with hypertriglyceridemia. They are moderately effective agents in reducing plasma TG (by 50%) and, to a lesser extent, LDL-C (≤20%), as well as in increasing HDL-C concentrations (≥20%) [127].

Concerning bone metabolism, preclinical data have demonstrated that fibrates and, in particular, fenofibrate promote *BMP-2* gene expression, thus, stimulating the osteoblast differentiation [128]. Fenofibrate has been shown to maintain FN and whole-body BMD and bone architecture in ovariectomized rats, compared with pioglitazone [129]. However, others have shown a detrimental effect on bone quality in mice with diabetes mellitus, through decreased collagen I and osteocalcin secretion, due to down-regulation of *Runx2* gene expression [130].

The evidence for any clinical effect of fibrates on bone health is generally poor. In a case-control study, including 124,655 fracture cases and 373,962 age- and gender-matched controls, an increased risk for non-statin lipid-lowering agents (mainly cholestyramine and fibrates) was demonstrated. In contrast to statins, the use of these non-statin drugs was associated with an increased crude risk of vertebral (OR 2.25; 95% CI 1.22–4.16) and total fractures (OR 1.14, 95% CI 1.00–1.30). However, this association lost significance after adjustment for potential confounders [131].

### 4.5. Omega-3 Fatty Acids

The omega-3 fatty acids (FA) and, in particular, docosahexaenoic (DHA) and eicosapentaenoic acid (EPA) are essential polyunsaturated FA, derived mainly from fish oil [127]. They are moderately efficacious in lowering serum TG concentrations in a dose-dependent manner, with usual doses of 2–4 g/day, although their effect on other lipoproteins is trivial [127]. A cardiovascular benefit has been shown in patients at very high CVD risk with high doses (2 g of EPA twice a day) [127].

Preclinical data suggest a protective effect of omega-3 FA on bone metabolism since a high dietary intake increases the rate of bone formation [132]. They also reduce osteoclastic activity and the ensuing bone resorption by 80%, as shown in rats fed with a purified diet rich in omega-3 FA [133]. Furthermore, fat-1 transgenic mice, which can convert omega-6 to omega-3 FAs, demonstrate significant acceleration in callus formation and fracture healing compared with controls [134].

Epidemiological data in humans regarding the effect of omega-3 FA on musculoske-letal outcomes have provided inconsistent results. A meta-analysis of observational stu-dies (including seven prospective and three case-control studies; *n* = 292,657 participants) showed an inverse association between fish consumption and the risk of hip fractures (pooled effect size 0.88, 95% CI 0.79–0.98, for the highest compared with the lowest quartile) [33]. However, in subgroup analysis, this association was evident only in case-control studies and in prospective studies with a sample size of ≥ 10,000 participants. Moreover, an inverse association between omega-3 FA intake and the risk of hip fracture was observed (pooled effect size: 0.89, 95% CI 0.80–0.99) [33].

In a systematic review and meta-analysis of ten RCTs published in 2012, a favorable effect of omega-3 FA on BMD or bone turnover markers was demonstrated in four studies, but only when co-supplemented with calcium, whereas three studies showed no effect. No data on fractures were available [135]. Another meta-analysis of 28 RCTs (23 studies on omega-3 FA; 0.4–5.8 g/day of EPA and/or DHA, 3.5–9.1 g/day of alpha-linoleic acid) showed no effect on LS (mean difference 0.03 g/cm^2^, 95% CI from −0.02 to 0.07) or FN BMD (mean difference 0.04 g/cm^2^, 95% CI from −0.00 to 0.07) (low or very low quality of evidence, respectively) [136]. A high omega-3 dose induced a slight increase in osteocalcin concentrations, but no effect was observed on other bone formation or bone resorption markers [136]. No data on fractures were available from both meta-analyses [135,136].

### 4.6. Niacin

Niacin (nicotinic acid) is effective in reducing serum TG concentrations by inhibiting the secretion of very-low-density lipoprotein (VLDL) particles from the liver [127]. It also increases HDL-C concentrations due to increased production of apolipoprotein-A1 in the liver [127]. However, its cardiovascular benefit has not been proven, and therefore, it is currently not available in Europe [127].

Very little data exist concerning the effect of niacin on skeletal outcomes. A prospe-ctive community-based study, the Cardiovascular Health Study (CHS), including 5187 men and women ≥ 65 years, showed a U-shaped association between dietary niacin consumption with hip fracture risk. In particular, both lowest (3.6–21.8 mg/day) and highest (41.0–102.4 mg/day) consumption were associated with increased risk of hip fracture [HR 1.31 (95% CI 1.04–1.66) and 1.53 (95% CI 1.20–1.95), respectively] compared with daily intakes of 21.9–40.9 mg. A trend for an inverse association with hip BMD was also found (*p* = 0.06) [137]. However, the latter was not confirmed in another study in 243 pre- and 137 postmenopausal Japanese women, showing a positive association between dietary niacin intake and calcaneus BMD [138].

### 4.7. Bile Acid Sequestrants

Very few data exist with regard to the effect of bile acid sequestrants, such as cholestyramine, colestipol, or colesevelam, on bone metabolism. In a prospective study, cholestyramine 24 g/day, administered either as monotherapy or in combination with pravastatin, had no effect on PTH, 25-hydroxy-vitamin D, and 1,25-dihydroxy-vitamin D concentrations [139].

## 5. Conclusions

Despite the high heterogeneity and the variable quality of evidence, dyslipidemia, mainly high TC and LDL-C and, to a lesser extent, TG concentrations, seems to be associated with low bone mass and increased fracture risk. This detrimental effect may be mediated directly through the increased oxidative stress and systemic inflammation that dyslipidemia is associated with, leading to increased osteoclastic activity and reduced bone formation, or through the atherosclerotic process, which affects bone’s vascularization. Other mechanisms, such as low estrogen, vitamin D and K status, and increased concentrations of PTH, homocysteine, and lipid oxidation products, may also contribute to this interplay. Regarding the effect of lipid-lowering therapy on bone metabolism, statins may slightly increase BMD, with a tendency to reduce fracture risk as shown in case-control and cohort studies, although available RCTs have not shown any effect of statins on fracture risk. This is also the case for omega-3 FA, whereas inconsistent or insufficient evidence exists for other commonly used lipid-lowering medications, such as ezetimibe, fibrates, and niacin. There is an exigent need for prospective, well-designed studies in males and females to elaborate on the putative association between lipids and bone strength.

## Figures and Tables

**Table 1:** Observational studies assessing the association between lipids and BMD in adults.

Author/Year	*n*	Country	Gender	Age (Years)	Association with BMD		
					TC/LDL-C	HDL-C	TG
Yamaguchi, 2002 [4]	214	Japan	F	47–86	-	+	no
Poli, 2003 [5]	1303	Italy	F	54.2 ± 4.3	-	no	N/A
Tankó, 2003 [6]	340	Denmark	F	50–75	-	N/A	N/A
Adami, 2004 [7]	982	Italy	M/F	35–82	+	-	+
Orozco, 2004 [8]	52	Spain	F	55.2 ± 3.8	-	-	-
Samelson, 2004 [9]	1162	USA	M/F	32–61	-	-	N/A
Cui, 2005 [10]	730	Korea	F	19–80	-	-	-
Solomon, 2005 [11]	13,592	USA	M/F	>17	no	no	no
Hsu, 2006 [12]	13,970	China	M/F	25–64	-	no	-
Dennison, 2007 [13]	513	UK	M/F	64	no	-	N/A
Tang, 2007 [14]	368	Taiwan	M	78	N/A	N/A	+
Makovey, 2009 [15]	497	Australia	F	20–81	-	-	no
Sivas, 2009 [16]	107	Turkey	F	45–79	no	no	no
Hernadez, 2010 [17]	289	Spain	M	63.8 ± 8.4	+	no	no
Go, 2012 [18]	958	Korea	F	58.6 ± 5.8	-	+	N/A
Pliatsika, 2012 [19]	591	Greece	F	53.0 ± 5.65	no	+	no
Kim, 2013 [20]	6300	Korea	M	19–85	-	-	-
Loke, 2018 [21]	1162	Taiwan	M/F	59.9 ± 7.3	no	+	no
Panahi, 2019 [22]	2426	Iran	M/F	69.1 ± 6.3	-	-	+
Chin, 2020 [23]	400	Malaysia	M/F	>40	N/A	no	no
Zhang, 2020 [24]	1116	China	F	58.2 ± 13.9	no	no	no

**Abbreviations:** BMD—bone mineral density; F—females; HDL-C—high-density lipoprotein cholesterol; LDL-C—low-density lipoprotein cholesterol; M—males; N/A—not available; no—no association; TC—total cholesterol; TG—triglycerides. **Notes:** (+) indicates positive association; (-) indicates negative association.

**Table 2:** Pathogenetic mechanisms linking dyslipidemia and atherosclerosis with impaired bone metabolism.

Direct effects	↑ Cholesterol → ↓ osteoblast differentiation, ↑ osteoclastogenesis ↓ HDL-C → ↓ osteoblast differentiation and function Oxidized LDL-C → ↑ bone loss ↑ Fat accumulation in the femoral head → ischemia and hypoxia ↑ Blood viscosity, which compromises bones’ blood supply
Estrogens	↓ Estrogens → ↓ osteoblast differentiation, ↑ osteoclastogenesis, ↓ bone mass, atherogenic dyslipidemia → ↑ atherosclerosis and fracture risk Inverse association between estrogen and serum homocysteine and oxidized LDL-C concentrations
Vitamin D, PTH	Low vitamin D status → secondary hyperparathyroidism → ↓ bone mass, dyslipidemia, ↑ cardiovascular risk
Inflammation	Dyslipidemia → systemic inflammation (↑ TNF-*α*, IL-1, IL-6, IL-17, C-reactive protein) → ↑ osteoclastogenesis, osteoporosis
Gla proteins (MGP and osteocalcin)	Involvement in mineralization of bones and arteries
Vitamin K	Essential co-factor for the formation of Gla proteins Protects against osteocalcin-induced calcification
Osteopontin	↑ Osteoclast activity, bone resorption ↑ Systemic inflammation, atherosclerosis, and plaque calcification
BMPs	Involved in osteoblast differentiation and proliferation Vascular calcification promotion
Homocysteine	↑ Osteoclastogenesis, osteoclast activity, bone resorption ↓ Blood supply and impairment of bone biomechanical properties Association with premature atherosclerosis and thromboembolism
Nitric oxide	↑ Vascular smooth muscle relaxation, ↓ LDL-C oxidation, platelet aggregation, and adhesion ↑ Bone formation and fracture healing
RANK/RANKL/OPG axis	↑ Osteoclastogenesis, osteoclast activity, bone resorption Association with arterial and valve calcification
Wnt pathway	Involved in intracellular cholesterol trafficking Regulation of osteoblastogenesis and bone formation

**Abbreviations:** BMPs—bone morphogenetic proteins; Gla—carboxyglutamic acid; HDL-C—high-density lipoprotein cholesterol; IL—interleukin; LDL-C—low-density lipoprotein cholesterol; MGP—matrix Gla protein, PTH—parathyroid hormone; RANK/RANKL/OPG—receptor activator of nuclear factor kappa-Β//RANK ligand/osteoprotegerin; TNF-α—tumor necrosis factor-α; Wnt—Wingless-related integration site; ↑: increased; ↓ decreased.

## Data Availability

Not applicable.
